# Live birth rate following a failed first in vitro fertilization cycle with no embryos for transfer

**DOI:** 10.1038/s41598-023-35221-5

**Published:** 2023-05-23

**Authors:** Xiaohui Dong, Xia Xue

**Affiliations:** Assisted Reproduction Center, Northwest Women’s and Children’s Hospital, Xi’an, China

**Keywords:** Biological techniques, Medical research

## Abstract

After a failed in vitro fertilization (IVF) procedure in which no transferable embryo was obtained, the possibility of a subsequent pregnancy for the patient is unknown. We conducted a cohort retrospective study evaluating the live birth rate in the subsequent cycles of the patients with no embryo for transfer in their first IVF attempt between 2017and 2020. The first cycle variables of patients who conceived in subsequent cycles were compared to those who did not. Additionally, for patients who conceived at last, variables related to ovarian stimulation were compared between the first cycle and the conceiving cycle. In accordance with the inclusion criteria, 529 were enrolled during the study period, of which 230 had successful pregnancies and 192 gave birth to a live infant. Cumulative live birth rates (CLBR) per cycle and patient were 26% and 36% respectively. Moreover, 99% of the live births were obtained within the first three attempts, beyond six cycles, there was no pregnancy. Stimulating variables in the first cycle were not effective in predicting the likelihood of a patient's subsequent pregnancy. Overall, patients who did not have embryos available for transfer in the first cycle had a 36% chance of getting a live birth in subsequent attempts, and the cause of failure should be considered.

## Introduction

One of the two most critical elements for in vitro fertilization (IVF)success is the embryo. There may be no embryos available for transfer due to factors such as the heterogeneity of the patients in the ovarian stimulation protocol and laboratory operations^1^. Moreover, there is little literature concerning the patients who did not obtain any embryos for transfer in their first IVF procedure. In our center, the rate of having no embryos for transfer is approximately 4% on the whole. After an initial failure, 50% of patients choose to try again. Evidence suggests that after repeated IVF/ICSI cycles, live birth might be achieved eventually for certain groups ^2^.

Nonetheless, in China where IVF costs have not been fully covered by public resources, the utilization of IVF is on the basis of the family's financial situation. A significant number of families will consider pursuing the most economical option and avoiding wasteful spending. Additionally, trying again means a drain on physical energy, time, and social resources.

In this study, we intended to provide patients who had not yielded any available embryos for transfer in their first IVF cycle with realistic information regarding what outcomes might be expected if they elect to undergo another cycle.

## Materials and methods

This was a retrospective cohort study conducted in Xi'an, Shaanxi province, at the Reproductive Center of Northwest Women's and Children's Hospital. It was approved by the ethics committee of the Northwest Women’s and Children’s Hospital (number 2018002) and the informed consent was waived on account of its retrospective nature with the approval of the ethics committee of the Northwest Women’s and Children’s Hospital. We guaranteed that all research methods strictly adhered to the requirements and regulations outlined in the Declaration of Helsinki. Women attending our center for their first IVF or Intracytoplasmic sperm injection (ICSI) treatment from January 2017 to December 2020 were included. Inclusion criteria were women who: (1) had no embryos for transfer in their first cycle; (2) had done at least one subsequent cycle and had not achieved a pregnancy until all the embryos transferred; (3) or obtain a live birth or pregnancy in the subsequent cycle. The women included in the study were divided into two groups based on whether they had a live birth at the observation nodes we set up. The variables of the two groups of women in the first cycle were compared, like maternal age, number of oocytes retrieval, Gn amount, estrogen (E2) and luteinizing hormone (LH) level and so on.

Ovarian stimulation was started on the third day of menstruation using a combination of Gonadotropin-releasing hormone (GnRH) Agonist/Antagonist with recombinant follicle-stimulating hormone (FSH). Trigger with Human chorionic gonadotropin (hCG) was administrated when there were more than three follicles ≥ 18 mm in diameter. 36 h later, oocyte retrieval was performed by transvaginal ultrasound-guided vaginal fornix puncture.oocytes were collected and fertilized with the husband's sperm with IVF or ICSI in the laboratory. After 64 to 68 h of culture, morphological embryo assessment was performed according to the Edward RG’s criteria based on the number of blastomeres, homogeneous degree of blastomeres, and degree of cytoplasmic fragmentation: grade (I): the blastomeres are of equal size, transparent, and free of fragments; (II): the blastomeres are uneven in size, and the fragmentation rate is < 10%; (III): the blastomeres are uneven in size, and the fragmentation rate is 10–50%; (IV): blastomere size is uneven, fragmentation rate > 50%. Grade I II III were identified suitable for transferring into the uterus. Therefore, the absence of embryos for transfer indicated that not a single embryo met the transfer criteria.

In our center, single embryo transfer especially blastocyst transfer is preferred. The criterion for blastocyst culture is that two or more top quality day 3 embryos have been obtained. Fresh embryo transfer is performed except for those with physical abnormalities that make them unsuitable for transfer at that time. In a few cases, women older than 38 are allowed to transfer two sub-optimal day 3 embryos.

Patients were instructed to maintain a full bladder on the day of embryo transfer. Embryos were placed in the uterus using a catheter (Cook, Ireland) with the guidance of transabdominal ultrasound. To ensure the successful landing of the embryo, the mucus in the cervical os was wiped in advance with a cotton swab soaked in warm and humid saline. This procedure was conducted with the assistance of senior physicians, nurses, and laboratory specialists.

The primary outcomes were clinical pregnancy and live birth. The secondary outcomes were miscarriage. Clinical pregnancy referred to the presence of a gestational sac. Live birth was defined as giving birth at least one live birth (> 24 weeks gestation). A miscarriage was the termination of a pregnancy prior to 28 weeks.

The data of the first cycle and the subsequent cycles were collected and compared. The variable data obtained from conceiving cycles were compared with cycles in which no pregnancy was achieved. We divided the participants into eight groups (including abnormal development of embryos, ICSI low fertilization rate, ICSI fertilization failure, ICSI abnormal fertilization, IVF low fertilization rate, IVF fertilization failure, IVF abnormal fertilization and poor ovarian response) based on the reasons why there were no transferable embryos available in order to further investigate the linked determinants of live birth. And explored the relationship between these factors and the result. In addition, we divided the patients into six groups according to the cause of infertility (male factor, ovulatory dysfunction, tubal factor, female multifactor, poor ovarian response (POR), unexplained infertility). Final live birth rates of each group were calculated and compared.

SPSS 22.0 was utilized for data analysis. For the purpose of comparing continuous variables between the two groups, Mann–Whitney U test was used. Categorical variables were compared by chi-square test. To assess the impact of potential confounders on pregnancy outcomes, odds ratios and 95% confidence intervals were calculated using binary regression. *P* < 0.05 was considered statistically significant.

## Result

A total of 27,932 patients received 34,933 cycles during the study period, and 1019 first cycles did not obtain embryos for transfer. In accordance with the inclusion criteria, a total of 556 patients were enrolled. And after removing the missing information, a total of 529 patients entered the final study stage.

Baseline data, ovarian stimulation and embryo fertilization of the first IVF cycle were demonstrated in Table 1. The basic characteristics, the CPR and cumulative live birth rate (CLBR) of each subsequent cycle were shown in Table 2. During the study period, 230 women achieved clinical pregnancies and 192 of them resulted in successful live births. In the second attempt, the clinical pregnancy rate and the live birth rate reached 34% and 28%, respectively. The highest cumulative clinical pregnancy rate and cumulative live birth rate per cycle reached 34%, 28%. Similarly, the highest cumulative clinical pregnancy rate and cumulative live birth rate per patient were 43% and 36%. On the other hand, of the 529 patients included, 95 underwent the second cycle with the result of no embryo was obtained. At the same time, 15 patients had experienced at least 2 cycles without embryos. Out of the 15 patients, 2 ended up with live births.

**Table 1:** Baseline characteristics of patients undergoing their first cycle with no embryo for transfer.

Number of patients	529
Maternal age (years), mean ± SD	32.32 ± 5.28
Maternal BMI, kg/m^2^, mean ± SD	22.82 ± 3.48
Infertility duration (years)	3.93 ± 3.25
No. of oocyte retrieved (n)	5.42 ± 4.36
Fertilization type (%)	
IVF	426
ICSI	103
Infertility type (%)	
Primary	299
Secondary	230
Basal LH, IU/L	5.35 ± 4.65
Basal FSH, IU/L	8.47 ± 4.37
Basal E2, pg/mL	71.46 ± 133.78
AFC	8.86 ± 5.76
Total Gn dose, IU	2579 ± 1041
endometrium thickness, mms	10.82 ± 2.87

Sperm characteristics	
	IVF ICSI
Concentration (10^6^/ml)	57.41 ± 29.60 32.37 ± 30.59
Motility (%)	53.96 ± 15.48 29.15 ± 23.18

**Table 2:** Reproductive outcomes of patients stratified by cycle rank.

Cycle rank	1	2	3	4	5	6	7	8	9	10
Patients	529	529	133	54	20	10	5	1	1	1
Number of clinical pregnancy	0	180	36	10	2	1	1	0	0	0
Number of live birth	0	155	27	8	1	0	1	0	0	0
Clinical pregnancy	0	180/529	36/133	10/54	2/20	1/10	1/5	0	0	0
%	0	34%	27%	19%	10%	10%	20%	0	0	0
Live birth	0	155/529	27/133	8/54	1/20	0	1/5	0	0	0
%	0	28%	20%	15%	5%	0	20%	0	0	0
Cumulative clinical pregnancy (cycle)	0	180/529	216/662	226/716	228/736	229/746	230/751	230/752	230/753	230/754
%	0	34%	33%	32%	31%	31%	31%	31%	31%	31%
Cumulative live birth (cycle)	0	155/529	182/662	190/716	191/736	191/746	192/751	192/752	192/753	192/754
%	0	28%	27%	27%	26%	26%	26%	26%	26%	26%
Cumulative clinical pregnancy (patients)	0	180/529	216/529	226/529	228/529	229/529	230/529	230/529	230/529	230/529
%	0	34%	41%	43%	43%	43%	43%	43%	43%	43%
Cumulative live birth (patients)	0	155/529	182/529	190/529	191/529	191/529	192/529	192/529	192/529	192/529
%	0	28%	34%	36%	36%	36%	36%	36%	36%	36%

Subsequently, for the patients who conceived or not ultimately, their basic characteristics and ovarian stimulation variables were compared (Table 3). We can conclude that the patients who get pregnant at last have a younger age (31.06 ± 4.45 vs 33.29 ± 5.66, *P* < 0.001), shorter years of infertility (3.48 ± 2.38 vs 4.27 ± 3.76, *P* < 0.001), less Gn amount (2471.97 ± 1005.02 vs 2532.89 ± 1188.95,* P* = 0.01), and lower E2 (2399.08 ± 2144.44 vs 2527.66 ± 2416.90, *P* = 0.02) as well as LH (2.66 ± 3.60 vs 3.88 ± 6.10,* P* < 0.001) levels on trigger day compared with those who do not in the first cycle.

**Table 3:** The ovarian stimulation characteristics in the first cycle of the patients who conceived and who did not.

	Pregnancy	No pregnancy	*P-*value
Number of patients	230	299	
Duration of infertility, years	3.48 ± 2.38	4.27 ± 3.76	< 0.001
Maternal age (years), mean ± SD	31.06 ± 4.45	33.29 ± 5.66	< 0.001
Number of oocyte retrieved	5.53 ± 4.13	5.34 ± 4.55	0.16
Mean endometrium thickness, mms	11.09 ± 2.92	10.44 ± 3.11	0.10
Gn amount, IU	2471.97 ± 1005.02	2532.89 ± 1188.95	0.01
Gn duration, days	10.33 ± 3.50	9.77 ± 3.50	0.91
E2 on trigger day, pg/mL	2399.08 ± 2144.44	2527.66 ± 2416.90	0.02
LH on trigger day, IU/L	2.66 ± 3.60	3.88 ± 6.10	0.002

**Figure 1:**
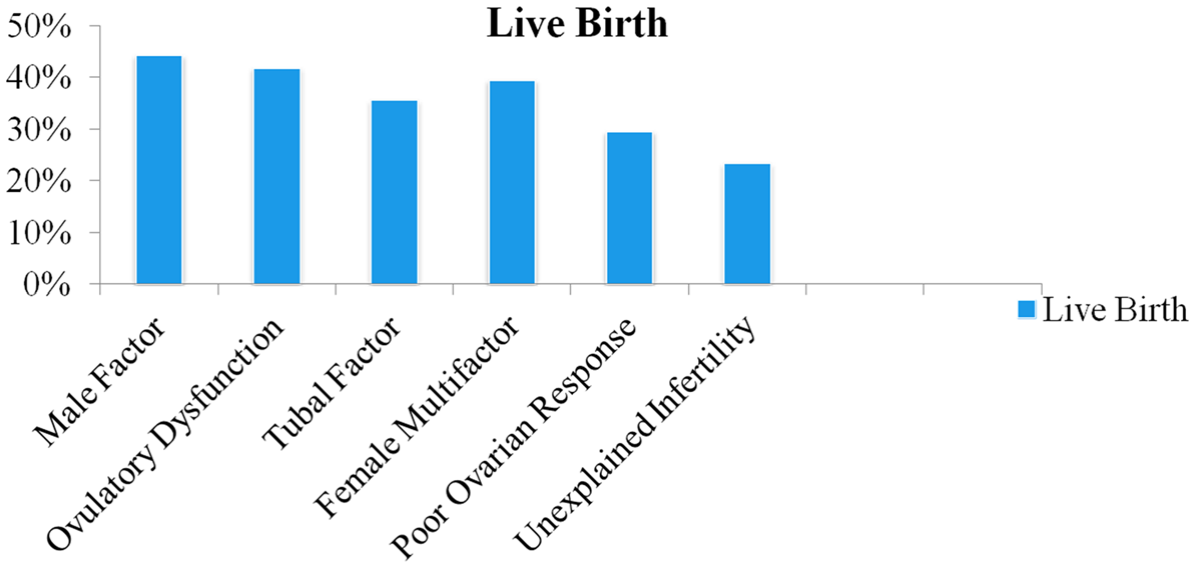
Comparison of live birth rate of the 6 groups when patients were grouped according to the cause of infertility.

The 529 enrolled patients were divided into six groups according to the cause of infertility: male factor, ovulatory dysfunction, tubal factor, female multifactor, POR, unexplained infertility. There were 77, 72, 203, 71, 61, 43patients in the groups respectively. The live birth rates of each group were calculated and shown in Fig. 1 according to the final outcome. For the couples with male factor, the final live birth rate reached 44.2%. While for the poor ovarian response group and unexplained infertility group, live births were harder to achieve (29.5%, 23.3% respectively).

We have made a comparison between the variables in the first and conceiving cycle of the patients who get pregnant at last (Table 4). Compared with the first cycle, there existed more oocytes (8.42 ± 4.89 vs 5.63 ± 4.12, *P* = 0.012) and starting Gn dose (244.06 ± 68.29 vs 213.41 ± 79.83, *P* = 0.004), less Gn duration (9.87 ± 2.21 vs 10.56 ± 3.18, P < 0.001) in the conceiving cycle.

**Table 4:** The ovarian stimulation characteristics of the first and conceiving cycle of the patients who conceived at last.

	First cycle	Conceiving cycle	*P*-value
Number of oocyte retrieved	5.63 ± 4.12	8.42 ± 4.89	0.012
Mean endometrium thickness, mms	11.26 ± 2.61	10.89 ± 2.73	0.272
Starting Gn dose, IU	213.41 ± 79.83	244.06 ± 68.29	0.004
Gn amount, IU	2526.66 ± 945.37	2601.76.53 ± 957.98	0.315
Gn duration, days	10.56 ± 3.18	9.87 ± 2.21	< 0.001
E2 (on trigger day), pg/mL	2441.49 ± 2135.68	2806.80 ± 2186.81	0.416
LH (on trigger day), IU/L	2.34 ± 2.07	2.63 ± 2.53	0.086

A logistic regression model was then used to assess the association between the reasons why there were no transferable embryos in the first cycle and final live birth while adjusting for potential confounders presented in Table 5. In the unadjusted model, the reason ICSI low fertilization rate was associated with live birth (OR 6.35, 95%CI1.29–31.22). After adjusting for covariates, the association still existed (OR 5.65, 95%CI 1.11–28.90).

**Table 5:** Odds ratios of the final live birth rates with reasons for not getting embryos for transfer in the first cycle.

Reasons		Live birth ultimately (%)	Crude model		Adjusted model	
			OR (95%CI)	*P* -value	OR (95%CI)	*P* -value
Abnormal development of embryos	119	46 (38.7)	Reference		Reference	
ICSI low fertilization rate	10	8 (80.0)	6.35 (1.29, 31.22)	0.023	5.65 (1.11, 28.90)	0.038
ICSI fertilization failure	8	3 (37.5)	0.95 (0.22, 4.18)	0.948	0.67 (0.14, 3.11)	0.609
ICSI abnormal fertilization	10	3 (30.0)	0.68 (0.17, 2.77)	0.590	0.53 (0.12, 2.34)	0.401
IVF low fertilization rate	61	28 (45.9)	1.35 (0.72, 2.51)	0.350	1.20 (0.63, 2.30)	0.581
IVF fertilization failure	160	49 (30.6)	0.70 (0.43, 1.15)	0.162	0.59 (0.33, 1.06)	0.079
IVF abnormal fertilization	5	0 (0)	0.00	0.999	0.00	0.999
poor ovarian response	156	55 (35.3)	0.86 (0.53, 1.42)	0.562	0.70 (0.38, 1.29)	0.249

Crude model: we did not adjust for other covariates. Adjusted model I: we adjusted for maternal age, AFC, number of oocyte retrieved and duration of infertility.

*OR* odds ratio, *CI* confidence interval.

## Discussion

To date, there were few reports regarding the subsequent clinical outcomes of patients who failed to obtain any transferable embryos during their initial attempt. The purpose of our study was to identify potential mechanisms affecting clinical pregnancy and live birth, and to provide some advice to patients who have failed their first IVF treatment. To our knowledge, our study's sample size is the largest of its kind for this topic. Our analysis indicated that the probability of subsequent live births was 36% for patients whose first IVF cycle resulted in no embryo for transfer.

One study investigated the question of the pregnancy predictive value of the stimulation characteristic in the first cycle in women of advanced age^3^. Different from ours, they thought the only statistically significant differences between women who achieved a clinical pregnancy and those who did not were maternal age and the number of oocyte retrieved. And we presume that shorter years of infertility, less Gn dosage and lower LH levels in the first cycle, rather than number of oocyte retrieved, predict a greater likelihood of pregnancy in the subsequent treatment.

Another study showed that extending cycles of IVF increased CLBRs and the increase was most evident during the first three cycles^2^. This conclusion is similar to ours, the majority of the patients can get live birth within three cycles.

Interestingly, we found that the CLBR of the patients in this study was similar to that of the POSEIDON 4 group or those with diminished ovarian reserve in the previous study^4–8^. This outcome may be attributable to infertility factors such as the patient's age. Orvieto et al.^9^ found that the factors predicting pregnancy or not were only maternal age and number of oocytes retrieved, different from ours. It was determined that age and ovarian response were the two most important factors, despite the fact that no indicators were found to predict future pregnancy from the first IVF attempt. The CLBR of the patients in this study was comparable to that of elderly patients in a previously published study where IVF treatment was prolonged up to 13 times^10^.

In this study, the levels of E2 and LH were lower in the first cycle than in the conceiving cycle. But the difference is not statistically significant. This was in line with some previous studies in which the researchers believed that low serum LH level and low serum E2 level on the day of HCG administration led to low clinical pregnancy rates^11–13^. However, other studies hold a different view concluding that there was insufficient evidence to support or deny the presence of the association^14,15^. It 's very interesting that we found that patients who conceived at last had lower levels of E2 and LH in the first cycle. Since the total amount of Gn in this group was also lower, we could speculate that this difference was caused by the amount of Gn used. At the same time, we can see that there were higher Gn initial dose and more oocyte retrieved in the conceiving cycle. For patients with normal ovarian response, the chance of pregnancy is increased by increasing the amount of Gn and the number of oocyte retrieved.

We discovered that out of the eight reasons for failure, ICSI low fertilization patients had more live births when patients were further categorized according to the cause of failure in the first cycle. It is noteworthy that the majority of patients with live births had oocyte activation agents used in subsequent cycles to help improve fertilization and embryo quality. Despite the modest size and limited scope of this group, it can still provide us with some useful information. Additionally, this supports some literature reports on the significance of oocyte activation^16,17^. In addition, we observed that patients with IVF low fertilization in the first cycle also resulted in higher live birth rates. This is not difficult to understand because the subsequent convention to ICSI can significantly improve the fertilization rate and the chance of live birth.

When it comes to infertility factors, it can be seen that the groups of male factor, ovulatory dysfunction, tubal factor had better live birth rates at the end. The odds of POR group and unexplained infertility group were pretty low. There is a clear association between POR and lower live birth rates^7^. When a POR patient ends up with no embryo for transfer, the situation will get worse.

Our current work has some notable strengths. In the first place, this is the first study with the largest sample size to date to explore possible future outcomes for patients who did not obtain embryos available for transfer in the first cycle. Additionally, the nature of retrospective studies may hinder observation bias. Last but not least, our research is based on actual clinical data, not a clinical trial, thereby avoiding the strict inclusion and exclusion criteria that may limit the representation and veracity of randomized controlled trials.

The limitations of this study should also be considered. First, due to the retrospective nature of our study, we were unable to control for potential unknown confounding factors. Then, since all of this data comes from one IVF treatment center, a larger multicenter study is needed to confirm the results.

In conclusion, our findings revealed that patients who did not obtain embryos available for transfer in their first cycle still had a 34% chance of conceiving in subsequent cycles, and the cause of failure should be considered at the same time.

## Data Availability

The datasets used and/or analysed during the current study are available from the corresponding author on request.
